# Transepidermal water loss rises before food anaphylaxis and predicts food challenge outcomes

**DOI:** 10.1172/JCI168965

**Published:** 2023-08-15

**Authors:** Charles F. Schuler, Kelly M. O’Shea, Jonathan P. Troost, Bridgette Kaul, Christopher M. Launius, Jayme Cannon, David M. Manthei, George E. Freigeh, Georgiana M. Sanders, Simon P. Hogan, Nicholas W. Lukacs, James R. Baker

**Affiliations:** 1 Division of Allergy and Clinical Immunology, Department of Internal Medicine,; 2 Mary H. Weiser Food Allergy Center,; 3 Michigan Institute for Clinical Health Research,; 4 Michigan Nanotechnology Institute for the Biomedical Sciences, and; 5 Department of Pathology, University of Michigan, Ann Arbor, Michigan, USA.

**Keywords:** Immunology, Inflammation, Allergy, Clinical practice, Diagnostics

## Abstract

**BACKGROUND:**

Food allergy (FA) is a growing health problem requiring physiologic confirmation via the oral food challenge (OFC). Many OFCs result in clinical anaphylaxis, causing discomfort and risk while limiting OFC utility. Transepidermal water loss (TEWL) measurement provides a potential solution to detect food anaphylaxis in real time prior to clinical symptoms. We evaluated whether TEWL changes during an OFC could predict anaphylaxis onset.

**METHODS:**

Physicians and nurses blinded to the TEWL results conducted and adjudicated the results of all 209 OFCs in this study. A study coordinator measured TEWL throughout the OFC and had no input on the OFC conduct. TEWL was measured 2 ways in 2 separate groups. First, TEWL was measured using static, discrete measurements. Second, TEWL was measured using continuous monitoring. Participants who consented provided blood samples before and after the OFCs for biomarker analyses.

**RESULTS:**

TEWL rose significantly (2.93 g/m^2^/h) during reactions and did not rise during nonreacting OFCs (–1.00 g/m^2^/h). Systemic increases in tryptase and IL-3 were also detected during reactions, providing supporting biochemical evidence of anaphylaxis. The TEWL rise occurred 48 minutes earlier than clinically evident anaphylaxis. Continuous monitoring detected a significant rise in TEWL that presaged positive OFCs, but no rise was seen in the OFCs that resulted in no reaction, providing high predictive specificity (96%) for anaphylaxis against nonreactions 38 minutes prior to anaphylaxis onset.

**CONCLUSIONS:**

During OFCs, a TEWL rise anticipated a positive clinical challenge. TEWL presents a monitoring modality that may predict food anaphylaxis and facilitate improvements in OFC safety and tolerability.

## Introduction

Food allergy (FA) is a substantial health burden affecting up to 10% of adults and 8% of children in the United States (1–3). FA causes food anaphylaxis, leading to 200,000 US emergency room visits yearly (4–6). FA diagnosis relies heavily on history because noninvasive diagnostics such as food-specific skin and blood IgE testing give positive predictive values as poor as 50% and do not predict anaphylaxis severity (7–9).

Oral food challenges (OFCs) are the FA diagnostic standard but are risky and labor intensive. During OFCs, a patient ingests a possible food allergen and often experiences anaphylaxis (9–11). However, there are significant barriers to performing in-office OFCs for FA, limiting the use of this test in clinical practice and therapeutic development (12). One major barrier to more widespread utilization of OFCs is the risk of anaphylaxis from the challenge. Therefore, the development of an accurate, reliable anaphylaxis monitoring and/or prediction technique for OFCs is sorely needed. Prior attempts have been made to develop such a system, including the use of skin thermography, but these techniques have not entered widespread use, probably in part due to the stringent conditions that must be maintained for such techniques (13, 14).

Transepidermal water loss (TEWL) is a well-established measure of skin barrier permeability (15, 16). TEWL measures water efflux in grams of water eluted from the skin per square meter per hour (g/m^2^/h). Elevated baseline nonlesional TEWL values may be positively associated with FA status in the context of atopic dermatitis, perhaps reflecting the key role that cutaneous epithelial barrier permeability may play in predisposing individuals to FA (17, 18). During anaphylaxis, blood vessels dilate (19, 20), increasing cutaneous heat and water loss (21–23); recent data suggest that more severe anaphylaxis results in greater extravasation of serum fluid and protein (24). Together, these findings suggest that TEWL could be an effective anaphylaxis-monitoring method if it can capture dynamic skin changes in real time. While TEWL has been used broadly as a static measurement, there is a paucity of data on its potential capacity as a dynamic anaphylaxis predictor.

This study sought to define whether TEWL measurement can detect anaphylaxis during OFCs; this question was evaluated in conjunction with both clinical and biochemical markers of anaphylaxis. Furthermore, this study aimed to evaluate whether continuous TEWL (cTEWL) measurement during an OFC can predict anaphylaxis before it becomes clinically evident and thus predict anaphylaxis prior to clinical diagnosis.

## Results

Table 1 details the composition of the “static” TEWL (sTEWL) measurement cohort. sTEWL measurements were obtained from 125 OFCs. The mean age was 8.7 years. The group was 38% female, predominantly White, and included a high proportion of individuals with other atopic conditions, including atopic dermatitis (AD), asthma, and allergic rhinitis/rhinoconjunctivitis. Table 2 details the FA composition of this group. A broad array of FAs is represented, particularly milk, egg, peanut, tree nut, and sesame allergies. Baseline demographic characteristics were largely similar between groups.

**Figure 1:**
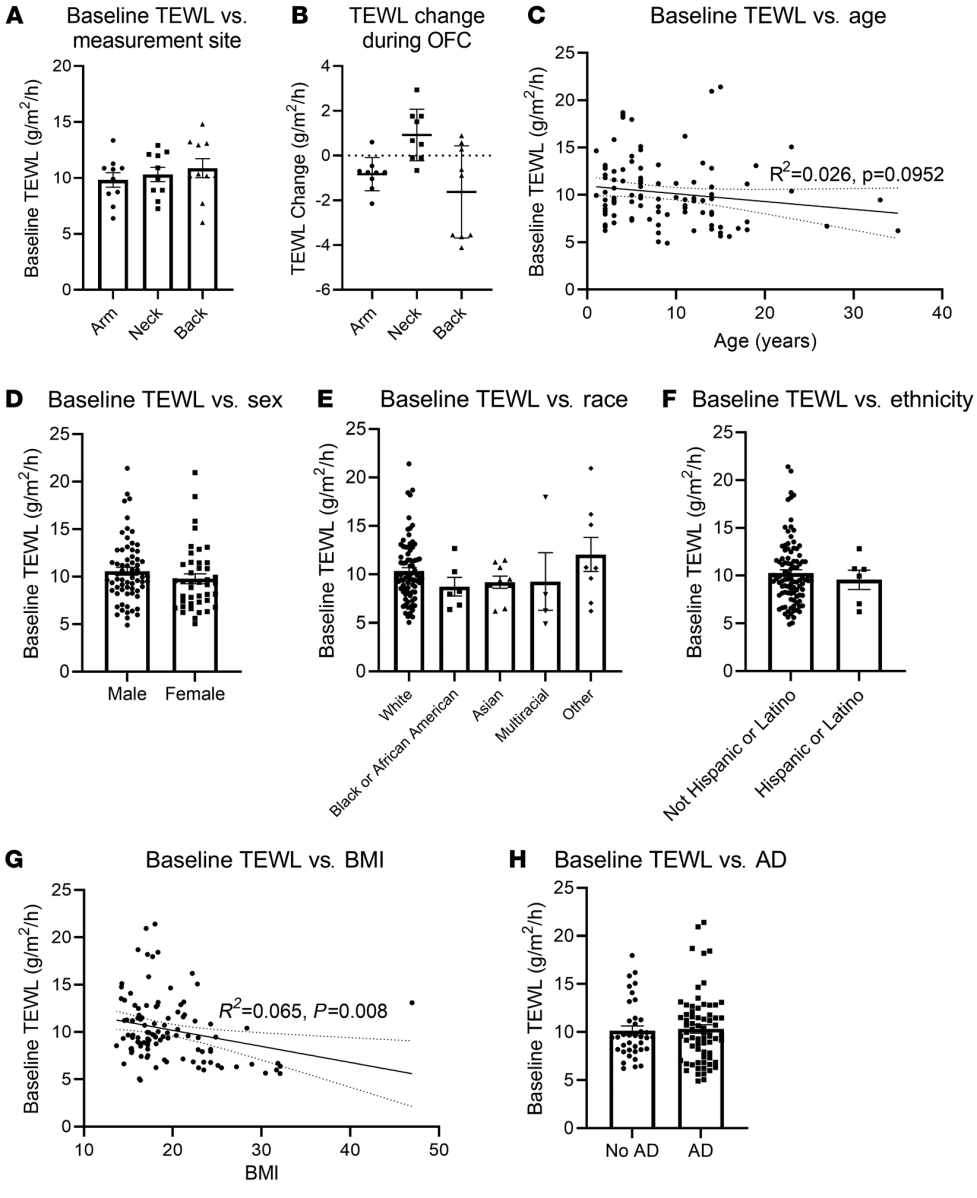
Influence of participants’ intrinsic characteristics on baseline TEWL. (**A**) Baseline TEWL results from 3 body areas (volar forearm, supraclavicular neck, upper posterior torso over the scapula). *n* = 10 per group. (**B**) Difference between baseline TEWL and TEWL at food dose 2 or 3 during the OFC. *n* = 9 per group. (**C**–**H**) Baseline TEWL on volar forearm shown by age (*n* = 107), sex (*n* = 107), race (*n* = 106), ethnicity (*n* = 105), BMI (*n* = 107), and AD status (*n* = 107). (**C** and **G**) Simple linear regression results with baseline static TEWL as the dependent variable and (**C**) age or (**G**) BMI as the sole independent variables.

In our initial studies, the location of the TEWL measurement was optimized to determine the most consistent method of data collection. We compared baseline TEWL measurements by body site in the first 10 nonreactive participants and found no significant difference between sites at baseline (arm mean = 9.82 g/m^2^/h; neck mean = 10.32 g/m^2^/h; back mean = 10.87 g/m^2^/h; *n* = 10 per group) (Figure 1A). The most consistent measurements were from the forearm, where measurements were stable and changed little, whereas measurements on the neck or back showed substantial variability and a notable change from baseline (Figure 1B). Given the ease of measuring TEWL on the forearm and these favorable measurement characteristics, we conducted the rest of the study using only forearm measurements. This approach is consistent with prior studies attempting to measure TEWL for atopic diseases (25, 26). Using forearm measurements, baseline TEWL decreased nonsignificantly with increasing age (*R^2^* = 0.026, *P* = 0.0952) (Figure 1C) but did not vary significantly by sex (Figure 1D), race (Figure 1E), or ethnicity (Figure 1F). A small decrease in TEWL was associated with a higher BMI (*R^2^* = 0.065, *P* = 0.008) (Figure 1G), and there was a trend toward a greater baseline TEWL among participants with AD (no AD mean = 10.15 g/m^2^/h; AD mean = 10.32 g/m^2^/h) (Figure 1H), but this was not significant. Thus, the forearm allowed consistent baseline data accumulation and was thereafter used to compare reacting versus nonreacting patient populations in this study.

**Figure 2:**
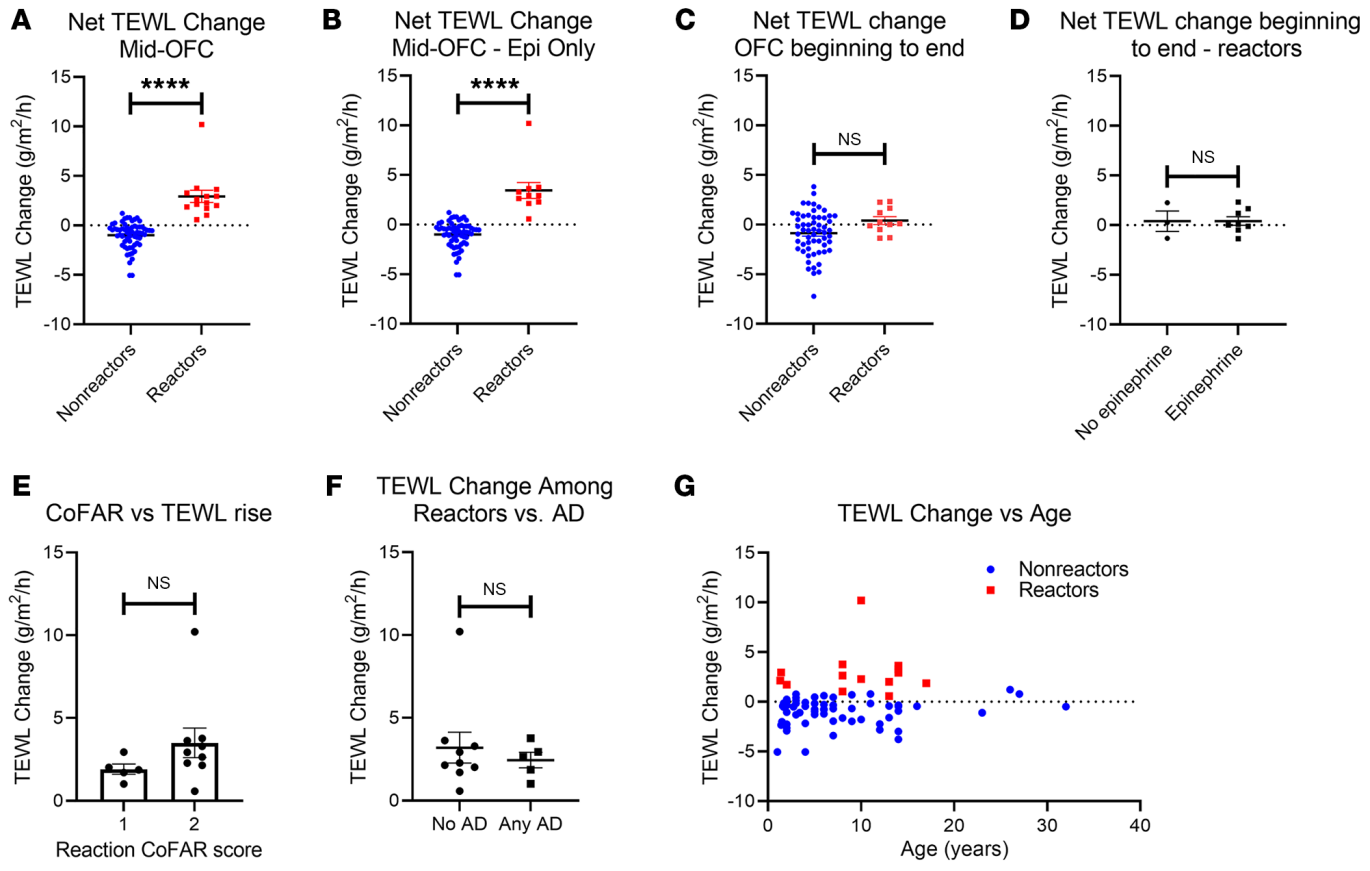
Change in sTEWL during OFC. (**A** and **B**) Difference between baseline TEWL and TEWL at food dose 2 or 3 (nonreactors, *n* = 62) or prior to epinephrine or other treatment (reactors) during the OFC. (**A**) All reactors (*n* = 14) and (**B**) only the reactors who required epinephrine (Epi) (*n* = 10). (**C**) Difference between baseline TEWL and TEWL at the end of the OFC (nonreactor, *n* = 58; reactor, *n* = 11). (**D**) Difference between baseline TEWL and TEWL at the end of the OFC for reactors only, separated according to participants who required epinephrine (*n* = 8) and those who did not (*n* = 3). (**E**) Difference between baseline TEWL and prior to epinephrine or other treatment versus CoFAR grade of anaphylaxis (*n* = 14). (**F**) Difference between baseline TEWL and prior to epinephrine or other treatment versus AD status (*n* = 14). (**G**) Difference between baseline TEWL and TEWL at food dose 2 or 3 (nonreactors, *n* = 62) or prior to epinephrine or other treatment (reactors, *n* = 14) during the OFC, shown by age and color coded for reaction status. Simple *t* tests were used to compare means for 2-variable plots. ****P < 0.0001.

**Figure 3:**
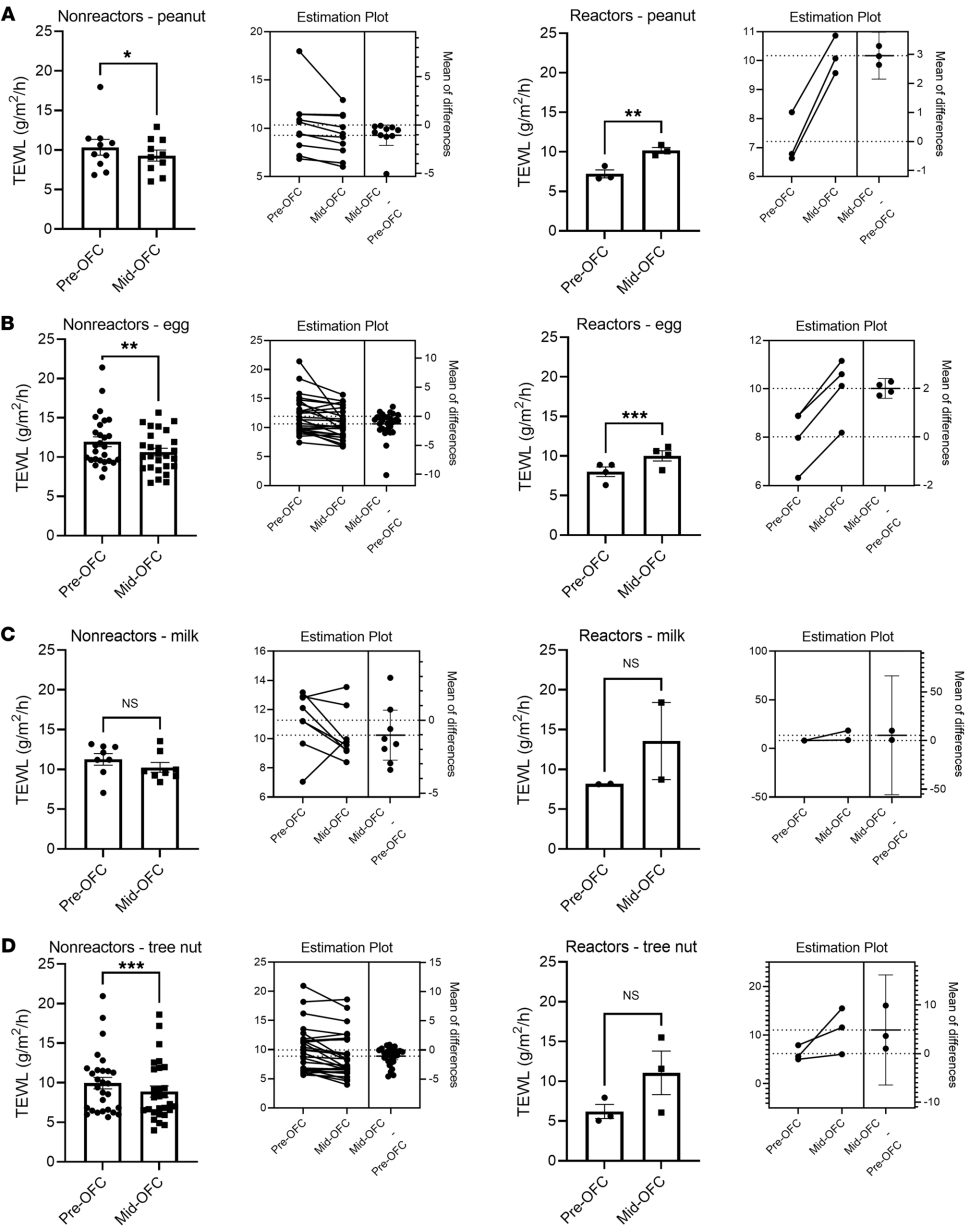
Change in sTEWL results by food type. Each panel shows the change in TEWL from baseline to either food dose 2 or 3 for nonreactors or prior to epinephrine or other treatment for reactors, with each panel delineated by food type. Estimation plots for pairwise *P* values for each group are shown. (**A**) Peanut OFCs (nonreactors, *n* = 10; reactors, *n* = 3). (**B**) Egg OFCs, which included both baked and cooked egg challenges (nonreactors, *n* = 27; reactors, *n* = 4). (**C**) Milk OFCs, which included both baked and unbaked milk OFCs (nonreactors, *n* = 8; reactors, *n* = 2). (**D**) Tree nut OFCs, which included all tree nut OFCs in the study (nonreactors, *n* = 28; reactors, *n* = 3). Paired *t* tests were used to compare means for 2-variable plots. *P < 0.05, **P < 0.01, and ***P < 0.001.

We then compared the change in TEWL obtained before the OFC started (baseline) with the value from the midpoint of the OFC (generally after food dose 3) for nonreactors and to the last measurement before anaphylaxis for reactors. Three placebo food challenges were included in the data set. We found that TEWL rose significantly during OFC reactions but was largely unchanged in the absence of a reaction (reaction mean increase = 2.93 g/m^2^/h vs. nonreaction mean increase = –1.00 g/m^2^/h, *P* < 0.0001) (Figure 2A). This phenomenon was present both when comparing the change in TEWL during reactions to nonreactions (Figure 2A) and when making a pairwise comparison of each individual’s baseline and mid-OFC TEWL values between reactors and nonreactors (Supplemental Figure 1). The rise in TEWL was also present when only severe reactions that required epinephrine were compared with nonreactors (reaction mean increase = 3.44 g/m^2^/h vs. nonreaction mean increase = –1.00 g/m^2^/h, *P* < 0.0001) (Figure 2B). TEWL values in patients with allergic reactions returned to levels similar to those of the unchanged nonreactors by the end of the challenge regardless of whether reactors received epinephrine (Figure 2, C and D). There was a trend toward a greater TEWL rise during Consortium for Food Allergy Research (CoFAR) grade 2 reactions (mean increase = 3.44 g/m^2^/h, *n* = 9), which required epinephrine, versus grade 1 reactions (mean increase = 1.91 g/m^2^/h, *n* = 5), which did not (Figure 2E), but this was not significant, and no reaction of grade 3 or higher occurred during the study period. The rise in TEWL noted during reactions was similar regardless of whether the participant had AD (Figure 2F) and across the age spectrum (Figure 2G). We further stratified the TEWL results by food. Four food groups, including peanut, egg, milk, and tree nuts, contained OFC reactions. The nonreacting peanut, egg, milk, and tree nut OFCs each demonstrated mean decreases in TEWL (peanut = –1.06 g/m^2^/h; egg = –1.32 g/m^2^/h; milk = –1.02 g/m^2^/h; tree nut = –1.07 g/m^2^/h), and all were statistically significant except for milk OFCs (Figure 3, A–D). The reacting peanut and egg OFCs demonstrated significant increases in TEWL (peanut = 2.96 g/m^2^/h; egg = 2.00 g/m^2^/h) (Figure 3, A and B), while the reacting milk and tree nut OFCs showed increases in mean TEWL that trended toward significance (milk = 5.39 g/m^2^/h; tree nut = 4.85 g/m^2^/h) (Figure 3, C and D). Estimation plots for pairwise comparisons are shown for each group in Figure 3.

Given that the TEWL changes documented here occurred on uninvolved skin (Figure 2, A and B), we also evaluated whether TEWL increases over the wheal or flare of urticarial lesions from resting baseline. We found that TEWL increased over both the wheal and the flare of histamine-induced hives as compared with baseline skin (baseline mean = 7.60 g/m^2^/h, flare mean = 10.19 g/m^2^/h, wheal mean = 11.54 g/m^2^/h) (Supplemental Figure 2). We also evaluated whether the observed increase in TEWL correlated with baseline food skin or blood IgE testing. We found that the degree of food sensitization defined by skin test wheal, skin test flare, or food-specific IgE did not show a significant correlation with the degree of TEWL change during OFCs (Supplemental Figure 3, A–F). Together, these data indicate that TEWL measurements correlated with the induction of a systemic anaphylactic response to OFC.

**Figure 4:**
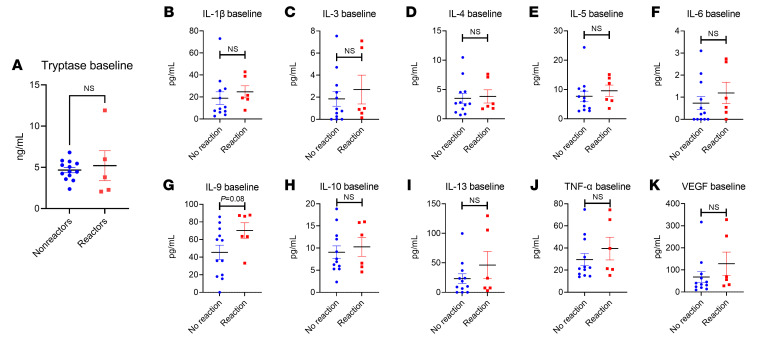
Baseline systemic immune markers. Baseline plasma results for (**A**) tryptase (*n* = 20), (**B**) IL-1β, (**C**) IL-3, (**D**) IL-4, (**E**) IL-5, (**F**) IL-6, (**G**) IL-9, (**H**) IL-10, (**I**) IL-13, (**J**) TNF-α, and (**K**) VEGF (*n* = 18 for **B**–**K**). All results are shown by reaction status. Simple *t* tests were used to compare 2-variable plots with normally-distributed data (tryptase, IL-1β, IL-5, IL-9), and Kruskal-Wallis tests were used to compare non-normally-distributed data (IL-3, IL-4, IL-6, IL-10, IL-13, TNF-α). Normality testing results are shown in Supplemental Table 1.

We sought to define whether the clinical reactions observed correlated with biochemical evidence of anaphylaxis. We therefore analyzed plasma from all participants that consented to give blood. We screened these samples for immune markers including tryptase, TNF-α, IL-1β, IL-3, IL-4, IL-5, IL-6, IL-9, IL-10, IL-13, and VEGF. At baseline, there were no significant differences between the reactor group and the nonreactor group in any marker tested (Figure 4, A–K). We did note a trend (*P* = 0.08) toward a greater IL-9 baseline systemic level among reactors (mean = 70.28 pg/mL) versus nonreactors (mean = 45.47 pg/mL), which may reflect prior studies indicating a role for elevated IL-9 activity in individuals with FA (27–29).

**Figure 5:**
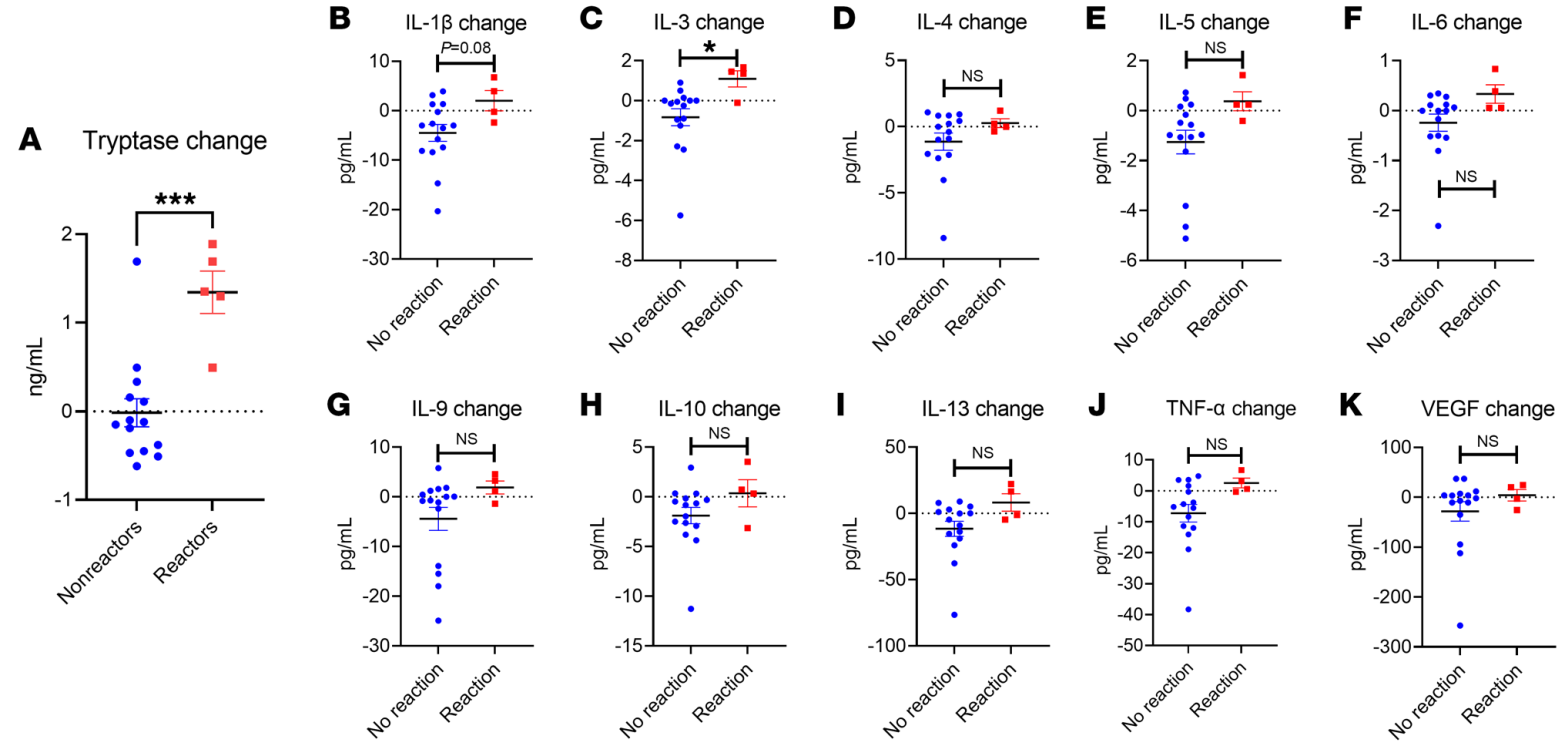
Change in systemic immune markers during OFC. Difference between post-OFC value and pre-OFC baseline result for the following markers: (**A**) tryptase, (**B**) IL-1β, (**C**) IL-3, (**D**) IL-4, (**E**) IL-5, (**F**) IL-6, (**G**) IL-9, (**H**) IL-10, (**I**) IL-13, (**J**) TNF-α, and (**K**) VEGF (all *n* = 19). All results are shown by reaction status. Simple *t* tests were used to compare 2-variable plots. *P < 0.05 and ***P < 0.001.

In contrast, we noted a significant increase in tryptase (mean increase = 1.34 ng/mL) and IL-3 (mean increase = 1.09 pg/mL) results compared with baseline in participants during clinical allergic reactions (Figure 5, A–K, and Supplemental Figure 4). In addition, the change in tryptase and the change in TEWL were significantly correlated with one another (Pearson’s *r* = 0.5915, *P* = 0.0076), whereas the changes in IL-3 and TEWL were not significantly correlated when analyzed together (Supplemental Figure 4). There were also nonsignificant trends toward increases in IL-1β, IL-6, and TNF-α in the participants with reactions, but no significant change in VEGF.

**Figure 6:**
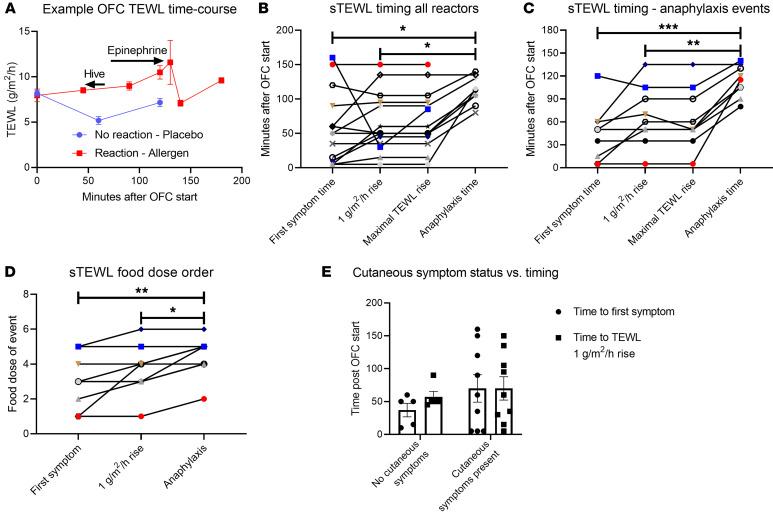
Timing of TEWL changes in relation to clinical events. (**A**) Representative time course of TEWL results for a reactive OFC (red) that led to anaphylaxis and a nonreactive challenge (blue). (**B** and **C**) Time to first symptom, to a 1 g/m^2^/h rise in TEWL, to the maximal TEWL rise, and to anaphylaxis (if it occurred) during OFCs resulting in (**B**) any reaction (*n* = 14) or (**C**) anaphylaxis that required epinephrine administration (*n* = 9). (**D**) Food dose of first symptom, of a 1 g/m^2^/h rise in TEWL, and of epinephrine administration during OFCs resulting in anaphylaxis and requiring epinephrine administration (*n* = 9). (**E**) Time to first symptom and to a 1-unit TEWL rise among reactors with cutaneous symptoms at any point (*n* = 5) or no cutaneous symptoms at any point (*n* = 9). Simple *t* tests were used to compare 2-variable plots, and ANOVA was used to compare means for plots showing 3 or more variables. *P < 0.05, **P < 0.01, and ***P < 0.001.

We sought to define the timing of the increase in TEWL observed among reactors as in Figure 2, A and B. We reviewed the timing of TEWL values for reactors during OFCs. Most reactions demonstrated a steady rise in TEWL from baseline, followed by a decrease after epinephrine (example in Figure 6A). We quantified the time to first symptom, the time to a TEWL rise of 1 g/m^2^/h from baseline, the time to the maximal TEWL change seen from baseline, and the time to epinephrine administration according to minutes after the start of the OFC and according to the food dose during which an event occurred. The time to first symptom (mean = 58.2 minutes) and the time to a 1-unit TEWL rise (mean = 65.4 minutes) were similar for most reactions, regardless of whether those reactions led to anaphylaxis (Figure 6B and Supplemental Figure 5). There was variability in whether the 1-unit TEWL increase or the first symptom occurred first from reaction to reaction. The time to a first symptom (mean = 44.4 minutes) and the time to a 1-unit TEWL rise (mean = 64.4 minutes) were each significantly less than the time to epinephrine (mean = 112.6 minutes) during anaphylaxis events (Figure 6C). Correspondingly, the food dose on which the first symptom or 1-unit TEWL rise also occurred earlier than the food dose of epinephrine administration (Figure 6D). The time to first symptom (no cutaneous symptoms mean = 37.0 minutes, cutaneous symptoms mean = 70.0 minutes) and the time to a 1-unit TEWL rise (no cutaneous symptoms mean = 57.0 minutes, cutaneous symptoms mean = 70.0 minutes) were not statistically different between individuals who produced any cutaneous symptoms (i.e., hives, angioedema, flushing) during their reactions and individuals who never produced a cutaneous symptom during their reactions (Figure 6E).

Given the early rise in TEWL noted here prior to anaphylaxis clinical diagnosis, we elected to evaluate whether cTEWL monitoring could predict clinical anaphylaxis. Table 3 defines the baseline characteristics of the individuals who had cTEWL measurement during the OFCs. The mean age was 13.5 years, which was older than the mean age of individuals in the sTEWL group. The group was 40% female and predominantly White and, again, included a large proportion of individuals with other atopic conditions. Table 4 details the FA composition of this group. Again, a broad array of FAs was represented. Baseline demographic characteristics were largely statistically similar between the reactor and nonreactor groups.

**Figure 7:**
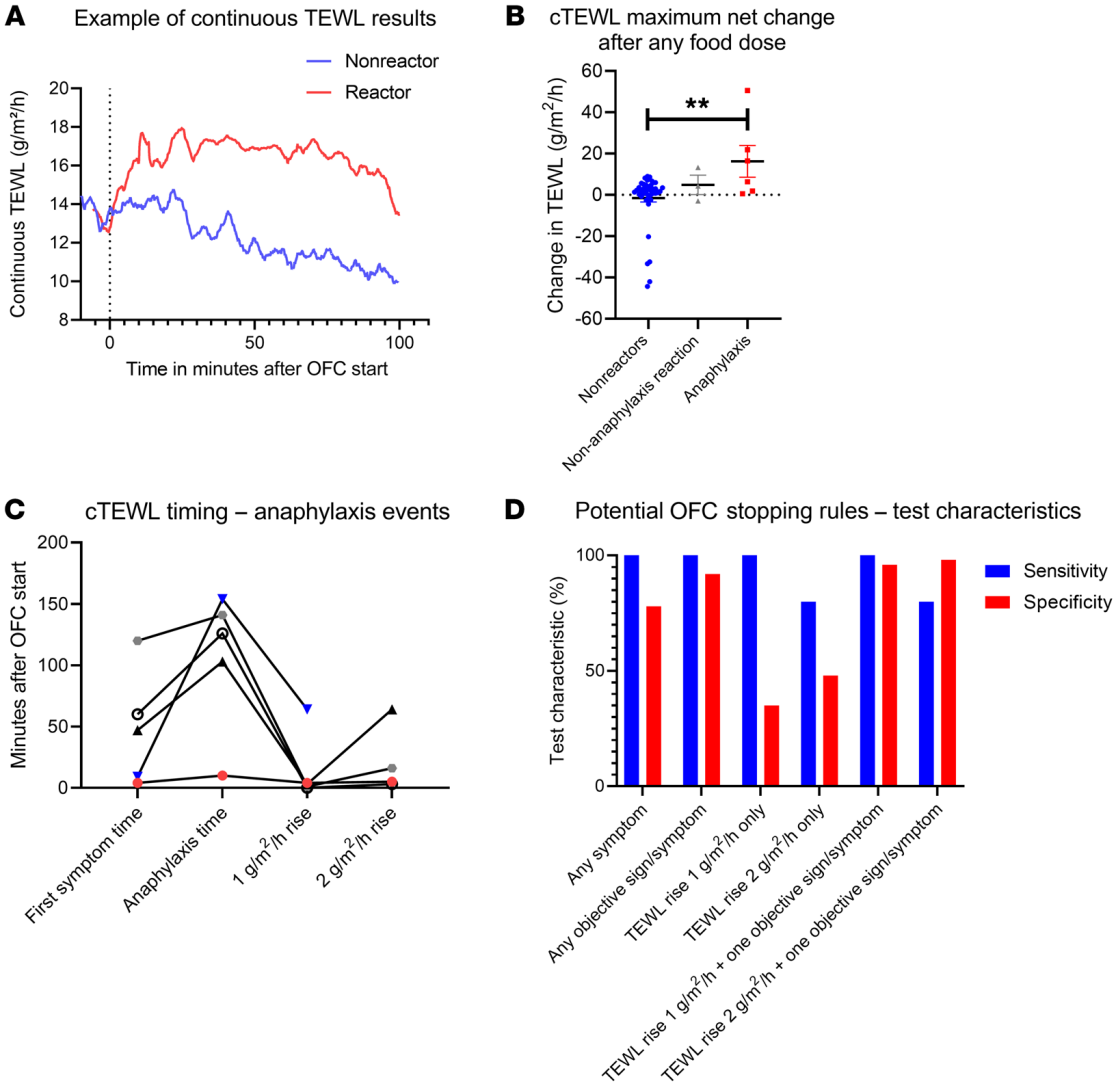
Results of cTEWL monitoring of the OFCs. (**A**) Example time course of a reactive OFC resulting in anaphylaxis (red) and a nonreactive OFC (blue). (**B**) The maximal net change in the mean TEWL value from the 2 minutes after any food dose during the OFC versus the 2 minutes prior to the same food dose (*n* = 53). (**C**) cTEWL event timing for anaphylaxis events in which the individual had a 1-unit TEWL increase, as in **B** (*n* = 5). (**D**) Sensitivity and specificity graphed for potential OFC stopping rules including for any symptom, any objective sign/symptom, 1- or 2-unit TEWL increases within 2 minutes before/after any food dose, and the 1- or 2-unit TEWL increases in combination with any objective symptom. ANOVA was used to compare means for plots showing 3 or more variables. **P < 0.01.

We evaluated the use of cTEWL monitoring in a subsequent group of individuals who underwent OFCs. Typical cTEWL results for individuals with and without allergic reactions are shown (Figure 7A). When taken together, the maximal net TEWL change after any food dose was significantly greater in the individuals with anaphylaxis than in the group of individuals who had no reaction or who had reactions without clinical anaphylaxis (Figure 7B). Among anaphylaxis reactions, a 1-unit TEWL increase generally occurred early during the reaction, in many cases prior to the time of the first symptom (first symptom mean = 48.0 minutes, 1 unit-TEWL increase mean = 14.4 minutes, anaphylaxis mean = 106.8 minutes (Figure 7C). On the basis of these results, we sought to define a TEWL change that would predict anaphylaxis before it was clinically evident. We used the time of epinephrine administration as a proxy for the onset of anaphylaxis and tested a variety of potential OFC stopping rules using these data. TEWL increases of 1 or 2 g/m^2^/h alone were not specific for anaphylaxis but did provide good sensitivity (100% for a 1-unit increase) (Figure 7D). Symptoms, whether subjectively reported or objectively adjudicated, indicated high sensitivity (100%) for anaphylaxis but also were not specific in isolation (Figure 7D). We should note that because the diagnosis of anaphylaxis requires that an individual have allergic symptoms/signs, the sensitivity value here reflects the definition of anaphylaxis, not a real predictive capacity. When we tested a potential stopping rule using the combination of any objective symptom/sign with a TEWL rise, we found that a 1 g/m^2^/h rise in TEWL plus any single objective symptom/sign of an allergic reaction produced 100% sensitivity and 96% specificity for impending anaphylaxis and provided an average of 38 minutes of warning prior to clinical anaphylaxis (Figure 7D).

## Discussion

These studies document that TEWL can be readily measured during OFCs and substantially increases during food allergy reactions and anaphylaxis. Furthermore, this rise correlated with biochemical markers of anaphylaxis and substantially preceded clinical detection of anaphylaxis. Using a monitoring-capable version of a commercial TEWL measurement device, we further show that TEWL monitoring offers a viable predictor of impending anaphylaxis well in advance of clinical reaction. Together, these observations suggest that TEWL may be useful as an anaphylaxis prediction method.

Given the high prevalence of FA worldwide (1–3) and the adverse effects associated with an inaccurate FA diagnosis, correct FA diagnoses are critical. The OFC remains central to diagnosis due to high false-positive rates for skin and blood food testing (7–9). It is interesting that the degree of TEWL changes observed in participants during this study correlated only with clinical reactivity and not with food-specific skin or blood IgE testing, suggesting that TEWL changes reflect clinical reactivity and may not have a relationship with IgE and related tests, which have well-known limitations (7–9).

Barriers to performing OFCs take multiple forms (12), and there is widespread interest in making this test safer and more accessible (7, 30). Prior attempts to use other methods to detect anaphylaxis in real time have not reached widespread use (13, 14). We suspect this is because facial thermography requires optics expertise, a specific, high-quality camera, tightly controlled ambient conditions (including temperature and cold lighting), and extremely compliant individuals willing to sit at a fixed distance from a camera for repeated image captures. Given that many OFCs for FA are performed in children, the potential limitations associated with participant behavioral nonadherence alone are obvious. TEWL is a simple measurement that can be readily replicated in offices without complex equipment. TEWL can also be affixed to the skin and provide meaningful data even among children, as seen here. This could provide a valuable enhancement of OFCs for clinical use, since the ability to detect and predict anaphylaxis prior to the need for epinephrine would improve the safety of this test.

The physiology of allergic reactions supports TEWL’s use for anaphylaxis prediction in the OFC. During anaphylaxis, blood vessel dilation increases cutaneous heat and water losses (19–23), suggesting a possible mechanism for TEWL’s ability to detect anaphylaxis in real time. Furthermore, recent data showing severe anaphylaxis results in greater extravasation of serum fluid and protein further support the use of TEWL measurement to monitor these reactions (24). In the present work, we note that the first symptom of a reaction generally occurred at a similar time to the noted TEWL changes (for example, Figure 5, B–E, and Figure 6C), supporting the concept that TEWL may correlate with early skin barrier or vascular fluid changes that occur during FA reactions. Further work in this area will define the molecular mechanism(s) by which TEWL changes during anaphylaxis.

TEWL monitoring has the potential to provide an anaphylaxis warning or prediction system. Combining cTEWL measurement with an objectively adjudicated sign/symptom of anaphylaxis has the potential to provide a high-specificity predictor of impending anaphylaxis. Clearly, avoiding false-positive results from TEWL monitoring (which would lead to false FA diagnoses) must be avoided, so the sensitivity/specificity analyses in Figure 6 must be interpreted with cautious optimism and should not be viewed as definitive. Indeed, there are cases in which nonreactor and reactor values overlap, so testing TEWL monitoring in a cohort at higher risk for anaphylaxis would be particularly useful to understand the true predictive power of this approach. Given the results presented here, the authors are conducting a pilot clinical trial deploying TEWL-based stopping rules during OFCs to determine whether the present data might be put into future testing and/or practice.

This study has limitations. First, most OFCs were not blinded. While unblinded OFCs are very commonly used and generally justified in clinical practice, foreknowledge of the food consumed could introduce confounding due to an expectation of reaction/nonreaction outcomes by the participants (31). In addition, the use of epinephrine as a surrogate marker for anaphylaxis could have led to an overestimate of the time from the TEWL rise to anaphylaxis. Second, because all participants were not required to give blood samples to participate, the results of the biomarker analysis could have limited power to detect true differences where none were seen. Third, all reactors in our sample set were under the age of 18 years, thus, we do not have the capacity to definitively comment on this phenomenon’s applicability to adults at the present time. Fourth, this study included a relatively limited number of reactions and only included reactions up to CoFAR severity grade 2; future work will be required to define the role of TEWL in more severe food anaphylaxis events. Fifth, blood samples were collected as soon as possible after a reaction; this could conceivably underrepresent the degree of changes among biomarkers such as tryptase, which may not have peaked at the time of collection.

In conclusion, changes in TEWL measurement occurred during OFCs prior to clinical evidence of allergic reactions and anaphylaxis. TEWL-based monitoring therefore deserves further evaluation as an anaphylaxis prediction tool. Prospective validation in clinical trials of TEWL-based OFC stopping rules could help define this utility. TEWL monitoring may also have implications in identifying other forms of anaphylaxis, which could provide further insight into the physiology underlying anaphylaxis.

## Methods

### Patient population and data collection.

This was an observational study involving patients undergoing OFCs in the Division of Allergy and Clinical Immunology at the University of Michigan from June 2021 through August 2022.

All participants underwent either an open OFC or a double-blinded, placebo-controlled OFC for management of an existing or suspected FA; a specific IgE or skin prick test cutoff was not enforced for enrollment. Clinical data were collected and managed using REDCap electronic data capture tools hosted at the University of Michigan (32).

### OFC protocol.

All OFCs were performed according to published guidelines (11, 31). Briefly, participants underwent clinical food-specific skin and blood IgE testing in addition to a thorough clinical FA history prior to OFCs. Participants were well on the date of the OFC, without infections, asthma, AD exacerbations, or recent anaphylaxis, per previously published guidelines (11, 33). Participants ingested escalating food doses every 15–20 minutes up to a full serving of the food challenged. Either 4 or 6 food doses were used for the OFCs, depending on the food challenged and total age-based food serving, per Table V and Figure 1 in Bird et al. (31). If no reaction occurred, participants were observed for at least 1 hour after the final food dose. If a reaction occurred, participants were assessed and treated by the attending allergist. Anaphylaxis was defined clinically by the attending allergist and treated with epinephrine, followed by other medications as deemed clinically appropriate by the treating allergist. Epinephrine was administered when the allergist diagnosed anaphylaxis. The clinical allergist could provide other treatment, such as an antihistamine dose, for a single symptom at their discretion. Anaphylaxis severity was graded by an independent allergist not present during the OFC, who reviewed the clinically documented OFC symptoms according to CoFAR criteria (34). All clinical events, including food doses, symptoms, and treatments, were recorded and time stamped by study staff.

### TEWL measurement methods.

For all TEWL measurements, the skin areas used were required to be clean and dry. All measurements were performed in 2 climate-controlled rooms. The ambient temperature and humidity were measured. Participants were given time to acclimatize to the room before measurements were taken. The clinical allergist, staff, and participant were blinded to the TEWL results throughout the OFC. The TEWL results were only linked to OFC data points after all OFC data were entered and the severity adjudicated.

Discrete or “sTEWL” measurements were taken using the Tewameter Hex device (Courage + Khazaka). Each measurement was taken in triplicate on the volar forearm (approximately one-third the distance from the wrist crease to the antecubital fossa, closer to the wrist), the anterior lower neck above the clavicle, or the upper back over the scapula. All measurements were taken on skin without visual evidence of rash, AD, hives, or other visual abnormalities. After the location-finding portion of the study, all TEWL measurements were taken on the volar forearm only, unless stated otherwise. When a reaction occurred, measurements were taken on visually normal, nonurticarial, nonflushed skin. Measurements during OFCs were taken just prior to ingestion of the first dose of food, between each food dose, as soon as possible during a reaction (if possible, without interfering with clinical care), immediately after epinephrine administration, and at the end of the waiting period after a challenge (whether a reaction occurred or not).

cTEWL values were measured using the Tewameter VT310 (Courage + Khazaka). The instrument was placed on the volar forearm using a 2-way adhesive. The instrument was in place prior to starting the OFC until the end but could be removed briefly as needed. These measurements were taken exclusively on skin that had no evidence of barrier disruptions, as above.

All TEWL measurements were recorded using MPA Plus software (Courage + Khazaka). Raw data were exported directly into Microsoft Excel (Microsoft) and analyzed as described below (see Statistics). All TEWL data points were time stamped to be collated with the clinical OFC data, including the times of the food doses, symptoms, and treatments.

### Biomarker analyses.

All participants who consented to biosample collection provided a blood sample immediately prior to the OFC and another sample either at the end of the observation period (in the case of no reaction) or as soon as possible after a reaction was identified, generally within 5 minutes, without interfering with clinical care (in the case of a reaction). Blood samples were collected into K2 EDTA vacutainers and immediately placed in 4°C and promptly separated into plasma by centrifugation. Plasma samples were aliquoted and immediately snap-frozen in liquid nitrogen until the time of analysis. Samples were not subjected to multiple freeze-thaw cycles.

The following analyses were performed using both pre- and post-OFC plasma samples. All kits were used according to the manufacturer’s instructions. The ImmunoCAP Tryptase fluoroenzyme immunoassay, analyzed on a Phadia 250 instrument (Thermo Fisher Scientific), was performed in the University of Michigan’s Clinical Laboratory Improvement Amendments–certified (CLIA-certified) clinical laboratory. Cytokines including TNF-α, IL-1β, IL-3, IL-4, IL-5, IL-6, IL-10, IL-13, and VEGF were analyzed using a customized cytokine/chemokine/growth factor immunology multiplex assay kit (MILLIPLEX, MilliporeSigma).

### Statistics.

All analyses were performed using GraphPad Prism (GraphPad Software) and SAS (SAS Institute) software. Descriptive statistics are provided for both the static and continuous samples stratified by reactors versus nonreactors using frequencies and percentages for categorical variables (χ^2^ for comparisons of reactors vs. nonreactors) and means, SDs, medians, IQRs, and ranges for continuous variables (and Kruskal-Wallis tests for comparisons). Normality testing was done for biomarker results using the Kolmogorov-Smirnov test, and non-normally-distributed group means were compared by Kruskall-Wallis test. TEWL and other inflammatory markers over time are presented in the figures using series, box, and bar plots. Comparisons of reactors versus nonreactors, where appropriate, were made with simple or paired *t* tests and ANOVAs. Linear regression models were also used to evaluate simple correlations between TEWL and characteristics such as age and BMI. A Pearson’s correlation was used to assess the correlation of tryptase and TEWL changes. Optimal thresholds for TEWL change in predicting a reaction from continuous data were determined by comparing maximum TEWL values before and after the first dose. We explored the predictive value of 1 or 2 g TEWL rises in predicting a subsequent reaction, and the change in predictive value when also requiring the presence of a reported symptom. Symptoms were categorized as subjective or objective, where subjective symptoms were unable to be verified by the examiner (i.e., mouth itching) and objective symptoms (or signs) were able to be verified by the examiner (i.e., a visible hive).

### Study approval.

All participants or their parent(s)/guardian(s) provided written informed consent for this study. Pediatric participants provided age-appropriate assent (assent was waived at age 6 and under). The study was performed in accordance with the Helsinki Declaration. The study protocol was approved by the University of Michigan Institutional Review Board under identifier HUM00165471.

### Data availability.

All data pertain to human participants in this study, and data requests will require a transfer agreement and should be directed to the corresponding author. Values for all data points in graphs are reported in the Supporting Data Values file.

## Author contributions

CFS, KMO, JPT, GMS, SPH, NWL, and JRB designed the research studies. JC and DMM conducted the laboratory analyses. CFS, KMO, BK, CML, JC, DM, GEF, and GMS contributed to data acquisition. CFS, KMO, JPT, BK, CML, JC, DMM, GEF, NWL, and JRB contributed to data analysis. All authors contributed to the writing and editing of the manuscript.

## Supplementary Material

Supplemental data

Supporting data values

## Figures and Tables

**Table 4:**
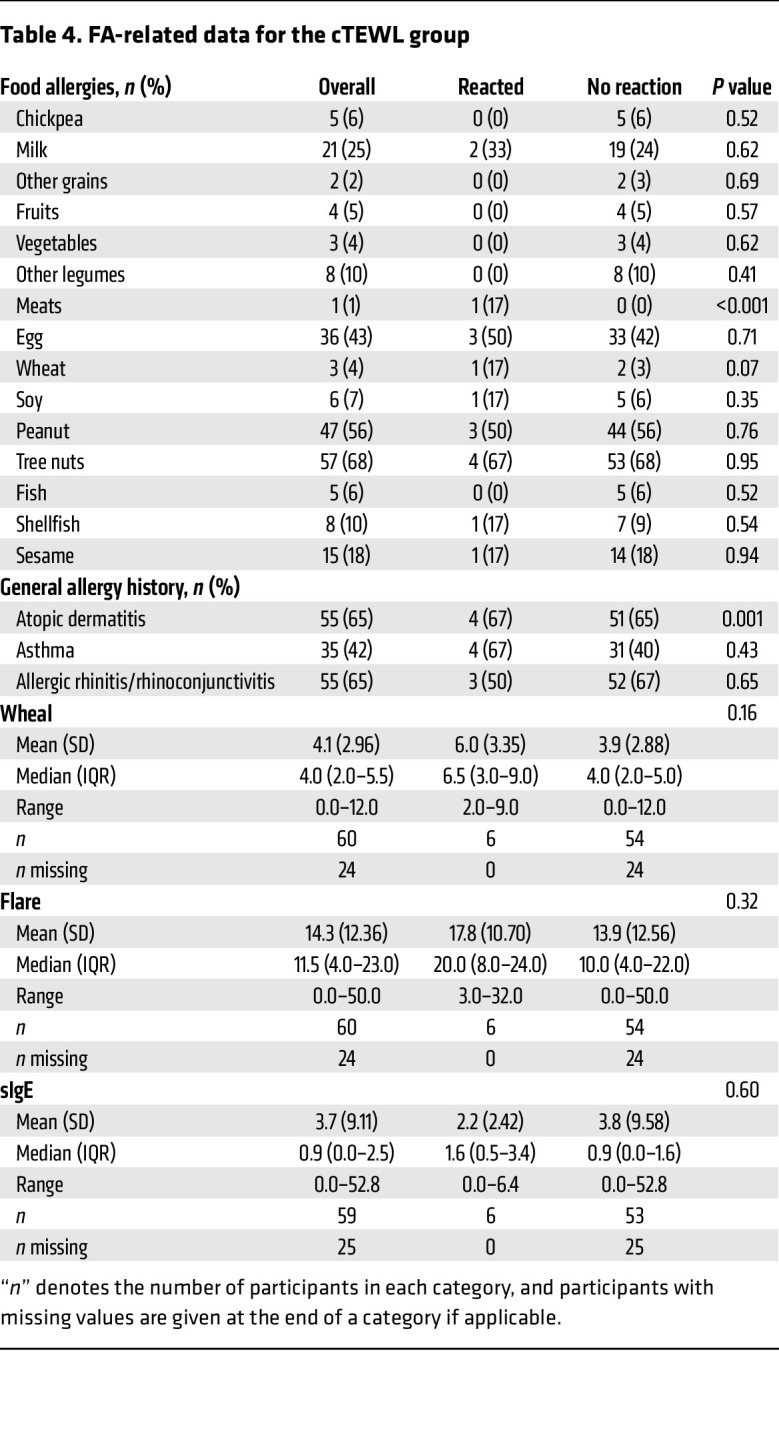
FA-related data for the cTEWL group

**Table 2:**
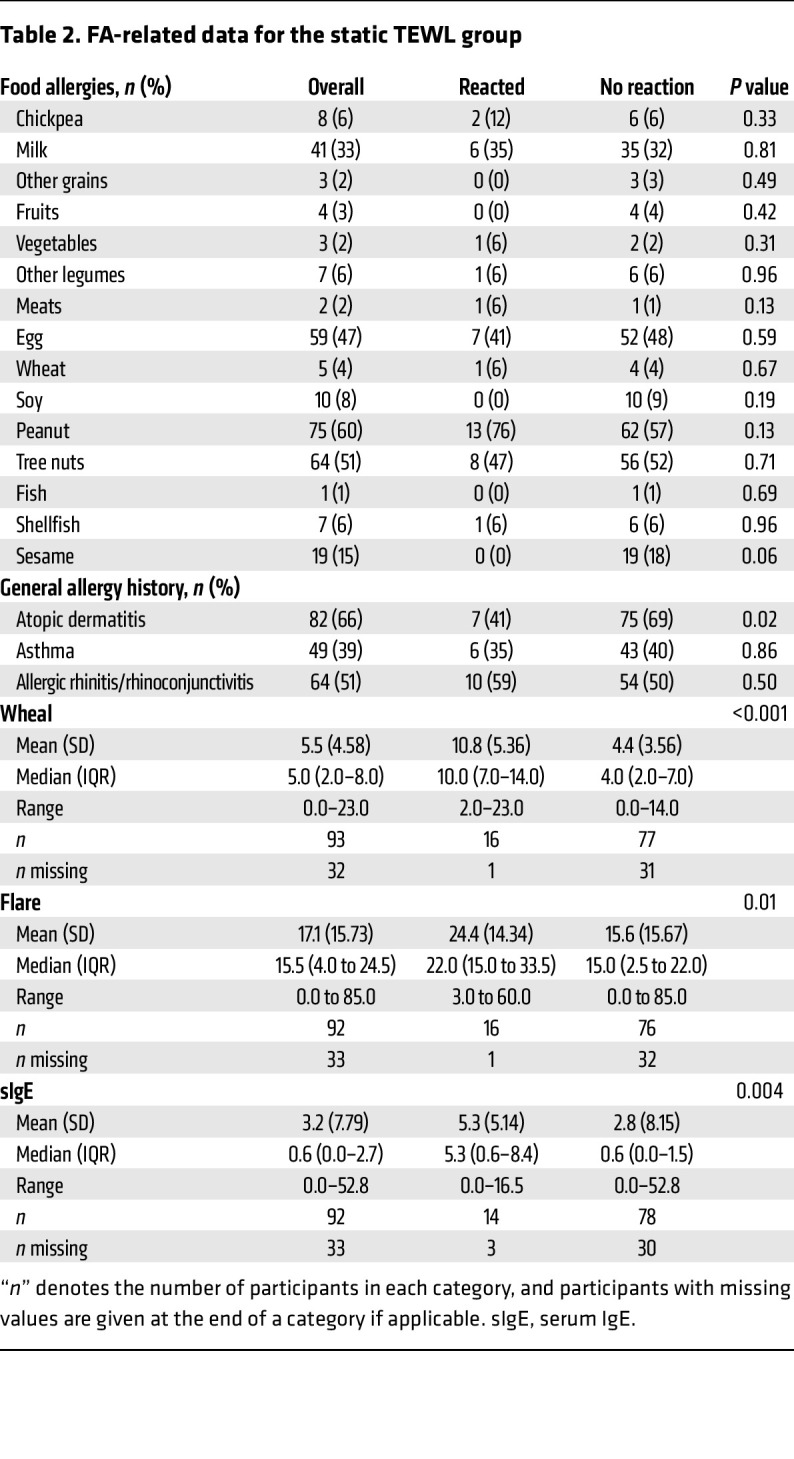
FA-related data for the static TEWL group

**Table 1:**
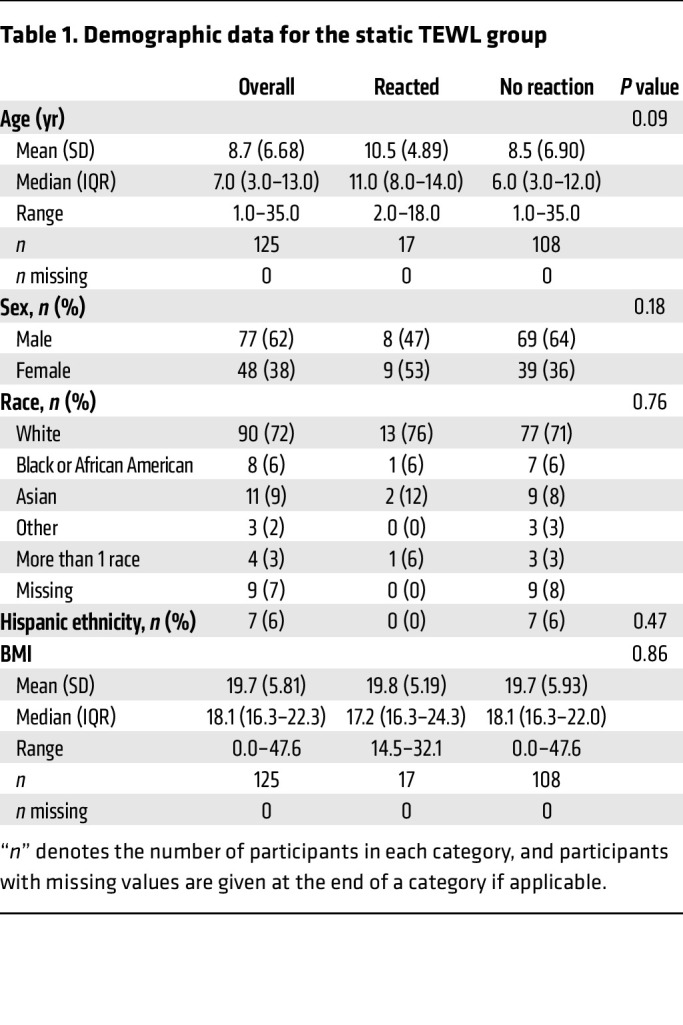
Demographic data for the static TEWL group

**Table 3:**
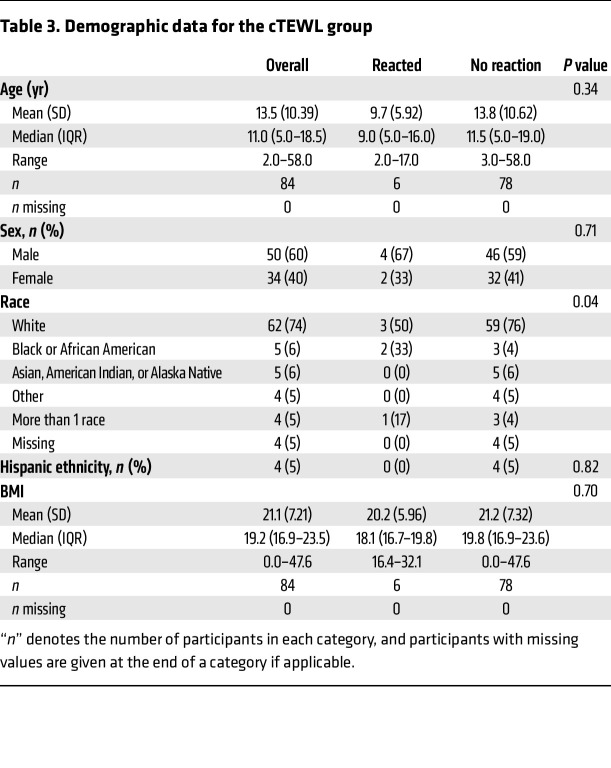
Demographic data for the cTEWL group

## Version 1 07/04/2023

In-Press Preview

## Version 2 08/15/2023

Electronic publication

## References

[1] Gupta RS (2018). The Public Health Impact of Parent-Reported Childhood Food Allergies in the United States. Pediatrics.

[2] Gupta RS (2019). Prevalence and severity of food allergies among US adults. JAMA Netw Open.

[3] Bilaver LA (2019). Economic burden of food allergy: a systematic review. Ann Allergy Asthma Immunol.

[4] Rudders SA (2010). Trends in pediatric emergency department visits for food-induced anaphylaxis. J Allergy Clin Immunol.

[5] Robinson LB (2021). Trends in US Emergency Department visits for anaphylaxis among infants and toddlers: 2006-2015. J Allergy Clin Immunol Pract.

[6] Sicherer SH (2011). Epidemiology of food allergy. J Allergy Clin Immunol.

[7] Koplin JJ (2019). Diagnosing peanut allergy with fewer oral food challenges. J Allergy Clin Immunol Pract.

[8] Peters RL (2013). Skin prick test responses and allergen-specific IgE levels as predictors of peanut, egg, and sesame allergy in infants. J Allergy Clin Immunol.

[9] Kawahara T (2019). Risk prediction of severe reaction to oral challenge test of cow’s milk. Eur J Pediatr.

[10] Calvani M (2012). Oral food challenge: safety, adherence to guidelines and predictive value of skin prick testing. Pediatr Allergy Immunol.

[11] Sampson HA (2014). Food allergy: a practice parameter update-2014. J Allergy Clin Immunol.

[12] Knibb RC (2012). The psychological impact of diagnostic food challenges to confirm the resolution of peanut or tree nut allergy. Clin Exp Allergy.

[13] Clark A (2012). Thermographic imaging during nasal peanut challenge may be useful in the diagnosis of peanut allergy. Allergy.

[14] Clark AT (2007). Facial thermography is a sensitive and specific method for assessing food challenge outcome. Allergy.

[15] Jansen van Rensburg S (2019). Measurement of transepidermal water loss, stratum corneum hydration and skin surface pH in occupational settings: A review. Skin Res Technol.

[16] Alexander H (2018). Research techniques made simple: transepidermal water loss measurement as a research tool. J Invest Dermatol.

[17] Leung DYM (2019). The nonlesional skin surface distinguishes atopic dermatitis with food allergy as a unique endotype. Sci Transl Med.

[18] Ashley SE (2017). The skin barrier function gene SPINK5 is associated with challenge-proven IgE-mediated food allergy in infants. Allergy.

[19] Triggiani M (2008). Allergy and the cardiovascular system. Clin Exp Immunol.

[20] Luo J (2021). Perioperative anaphylaxis from a perspective of temperature. J Invest Surg.

[21] Makabe-Kobayashi Y (2002). The control effect of histamine on body temperature and respiratory function in IgE-dependent systemic anaphylaxis. J Allergy Clin Immunol.

[22] Manzano-Szalai K (2016). Anaphylaxis imaging: non-invasive measurement of surface body temperature and physical activity in small animals. PLoS One.

[23] Kind LS (1955). Fall in rectal temperature as an indication of anaphylactic shock in the mouse. J Immunol.

[24] Nuñez-Borque E (2022). Personalized diagnostic approach and indirect quantification of extravasation in human anaphylaxis. Allergy.

[25] Perkin MR (2021). Association of frequent moisturizer use in early infancy with the development of food allergy. J Allergy Clin Immunol.

[26] Gerner T (2020). ‘Barrier dysfunction in Atopic newBorns studY’ (BABY): protocol of a Danish prospective birth cohort study. BMJ Open.

[27] Forbes EE (2008). IL-9- and mast cell-mediated intestinal permeability predisposes to oral antigen hypersensitivity. J Exp Med.

[28] Chen CY (2015). Induction of Interleukin-9-producing mucosal mast cells promotes susceptibility to IgE-mediated experimental food allergy. Immunity.

[29] Brough HA (2014). IL-9 is a key component of memory TH cell peanut-specific responses from children with peanut allergy. J Allergy Clin Immunol.

[30] Foong RX (2021). Improving diagnostic accuracy in food allergy. J Allergy Clin Immunol Pract.

[31] Bird JA (2020). Conducting an oral food challenge: an update to the 2009 adverse reactions to foods committee work group report. J Allergy Clin Immunol Pract.

[32] Harris PA (2019). The REDCap consortium: building an international community of software platform partners. J Biomed Inform.

[33] Greiwe J (2019). Oral food challenges in infants and toddlers. Immunol Allergy Clin North Am.

[34] Burks AW (2012). Oral immunotherapy for treatment of egg allergy in children. N Engl J Med.

