# Supplementary material for: Transepidermal water loss rises before food anaphylaxis and predicts food challenge outcomes
Source: J Clin Invest. 2023 Aug 15;133(16):e168965. doi: 10.1172/JCI168965 (PMC10425212; doi:10.1172/JCI168965)
Supplement: Supplemental data [file jci-133-168965-s027.pdf]

Supplemental Table 1: Normality testing for baseline biochemical markers

Biomarker	Test	p-Value
Tryptase	Kolmogorov-Smirnov	0.1500
IL_1b	Kolmogorov-Smirnov	0.1500
IL_3	Kolmogorov-Smirnov	0.0100
IL_4	Kolmogorov-Smirnov	0.0100
IL_5	Kolmogorov-Smirnov	0.1500
IL_6	Kolmogorov-Smirnov	0.0100
IL_9	Kolmogorov-Smirnov	0.0956
IL_10	Kolmogorov-Smirnov	0.0428
IL_13	Kolmogorov-Smirnov	0.0100
TNF_alpha	Kolmogorov-Smirnov	0.0100
VEGF	Kolmogorov-Smirnov	0.0140

Supplemental Figure 1: Change in sTEWL results from baseline – nonreactors vs reactors. Each panel shows the change in TEWL from baseline to either food dose 2 or 3 for nonreactors (n = 62) or prior to epinephrine or other treatment for reactors (n = 14). Estimation plots for pairwise p values for each group are shown. **** $p < 0.0001$. *** $p < 0.001$.

Supplemental Figure 2: TEWL values on normal skin versus urticarial wheal and flare. TEWL values on skin without a hive (normal skin, n = 3) and skin with a histamine-induced hive (flare n = 2, wheal n = 5). Each point represents a measurement taken in triplicate. “Normal skin” denotes skin without any visual evidence of any lesion from a volar forearm with no skin prick testing done. “Flare” measurements were taken from skin with a visible flare as soon as the flare was visible after a skin prick test histamine positive control was placed. “Wheal” measurements were taken from skin with a visible wheal as soon as the wheal was visible and measured > 3 mm after a skin prick test histamine positive control was placed. ANOVA was used to compare means for three-or-more-variable plots. ** $p < 0.01$. * $p < 0.05$.

Supplemental Figure 3: TEWL change correspondence with wheal and flare values of skin testing and blood IgE testing. Each panel shows the TEWL change plotted on the y-axis versus the relevant food test result, whether skin test wheal, skin test flare, or food-specific blood IgE result (total n = 84).

Supplemental Figure 4: Additional details on tryptase and IL-3 changes. Tryptase (n = 20) and IL-3 (n = 14) changes from baseline by reaction status are shown in the left panels. Tryptase and IL-3 changes are shown plotted against the challenge static TEWL change in the right panels. Pairwise t-tests were used to compare baseline to post-OFC for tryptase and IL-3 values. A Pearson correlation coefficient for TEWL change and biomarker change was calculated for each plot, denoted by Pearson r here. The Pearson r significance is denoted by the p value listed on the R panels. * $p < 0.05$. ns = not significant.

Supplemental Figure 5: Time to event for reactions delineated by epinephrine requirement. Time to first symptom and time to 1-unit TEWL rise for reactions requiring epinephrine (n = 9) and for reactions that did not (n = 5). Note that CoFAR grade 2 reactions were treated with epinephrine and CoFAR grade 1 reactions were not. Simple t-tests were used to compare two-variable plots. ns = not significant.

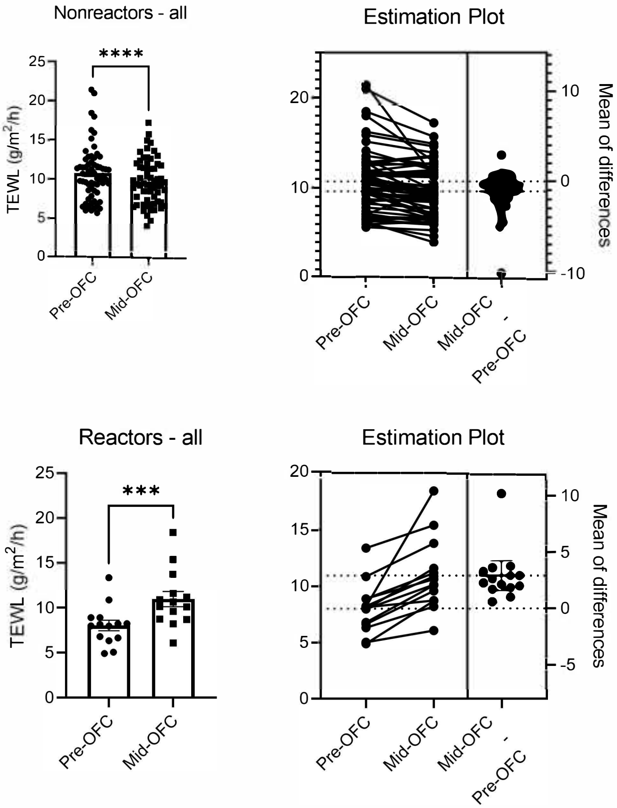

Nonreactors - all
****
TEWL (g/m²/h)
0
5
10
15
20
25
Pre-OFC
Mid-OFC
Estimation Plot
0
10
20
-10
0
10
Mean of differences
Pre-OFC
Mid-OFC
Mid-OFC - Pre-OFC
Reactors - all
***
TEWL (g/m²/h)
0
5
10
15
20
25
Pre-OFC
Mid-OFC
Estimation Plot
0
5
10
15
20
-5
0
5
10
Mean of differences
Pre-OFC
Mid-OFC
Mid-OFC - Pre-OFC

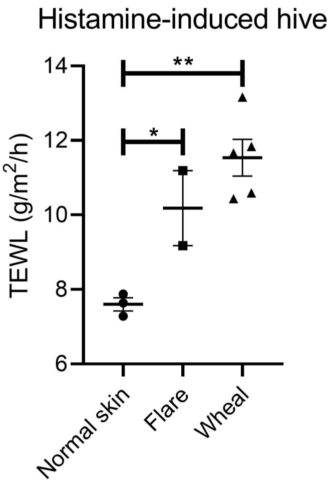
Histamine-induced hive
TEWL (g/m2/h)
14
12
10
8
6
**
*
Normal skin
Flare
Wheal

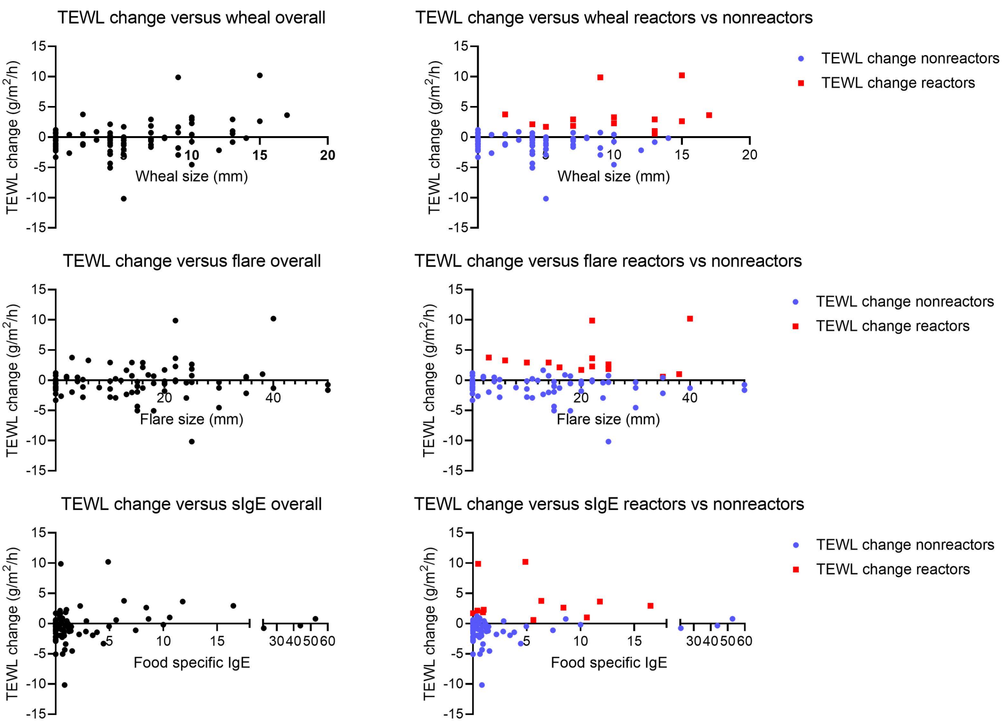
TEWL change versus wheal overall
TEWL change versus wheal reactors vs nonreactors
TEWL change (g/m2/h)
Wheal size (mm)
TEWL change nonreactors
TEWL change reactors
TEWL change versus flare overall
TEWL change versus flare reactors vs nonreactors
Flare size (mm)
TEWL change versus sIgE overall
TEWL change versus sIgE reactors vs nonreactors
Food specific IgE

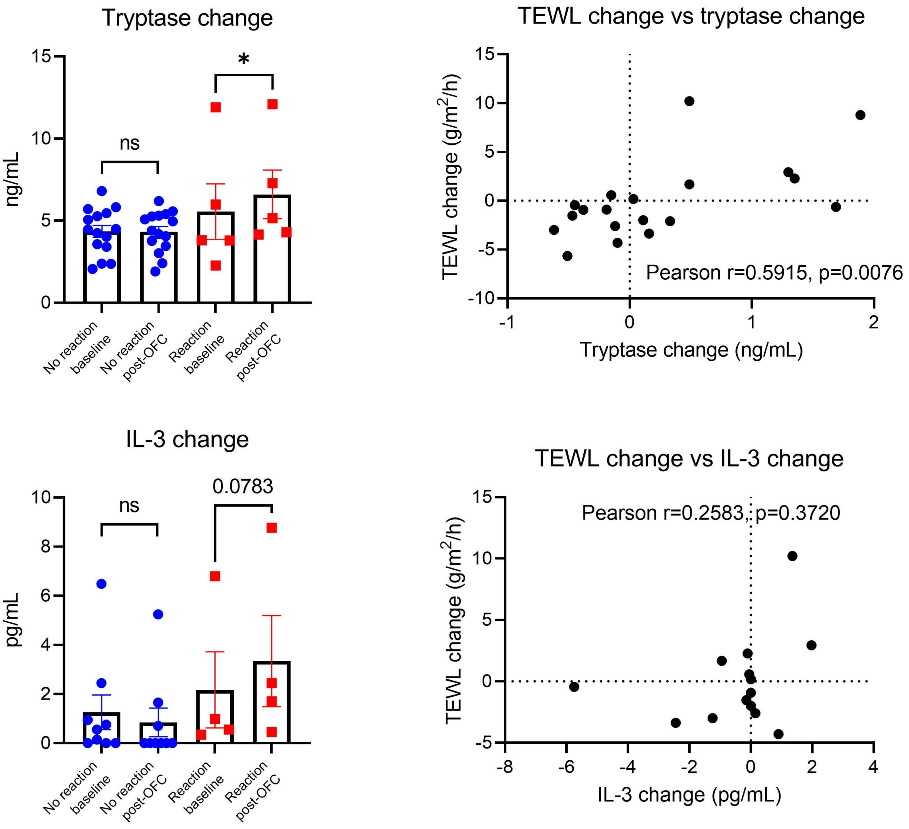
Tryptase change
ng/mL
15
10
5
0
ns
*
No reaction baseline
No reaction post-OFC
Reaction baseline
Reaction post-OFC
TEWL change vs tryptase change
TEWL change (g/m²/h)
Pearson r=0.5915, p=0.0076
Tryptase change (ng/mL)
-10
-5
-1
1
2
IL-3 change
pg/mL
8
6
4
2
ns
0.0783
TEWL change vs IL-3 change
Pearson r=0.2583, p=0.3720
IL-3 change (pg/mL)
-8
-6
-4
-2
4

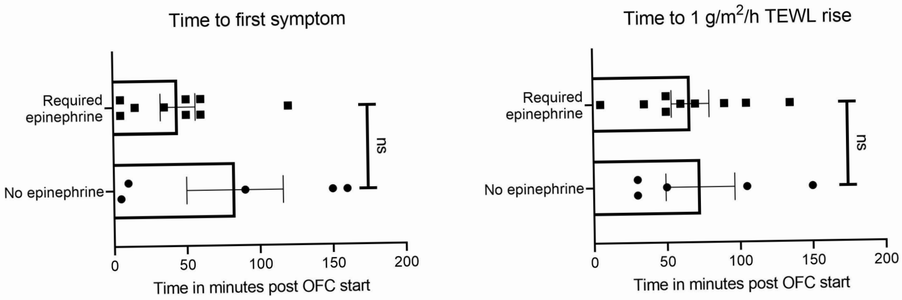

Time to first symptom
Required epinephrine
No epinephrine
ns
0
50
100
150
200
Time in minutes post OFC start
Time to 1 g/m2/h TEWL rise
Required epinephrine
No epinephrine
ns
0
50
100
150
200
Time in minutes post OFC start
